# New insights into apolipoprotein A5 in controlling lipoprotein metabolism in obesity and the metabolic syndrome patients

**DOI:** 10.1186/s12944-018-0833-2

**Published:** 2018-07-27

**Authors:** Xin Su, Yi Kong, Dao-quan Peng

**Affiliations:** 1 Department of Cardiovascular Medicine, the Second Xiangya Hospital, Central South University, Changsha, 410011 Hunan China; 2 Department of Dermatology, Hunan Key Laboratory of Medical Epigenomics, the Second Xiangya Hospital, Central South University, Changsha, 410011 Hunan China

**Keywords:** Apolipoprotein A5, Lipoprotein metabolism, Obesity, Metabolic syndrome, Adipocytes

## Abstract

Apolipoprotein A5 (apoA5) has been identified to play an important role in lipid metabolism, specifically in triglyceride (TG) and TG-rich lipoproteins (TRLs) metabolism. Numerous evidence has demonstrated for an association between apoA5 and the increased risk of obesity and metabolic syndrome, but the mechanism remains to be fully elucidated. Recently, several studies verified that apoA5 could significantly reduce plasma TG level by stimulating lipoprotein lipase (LPL) activity, and the intracellular role of apoA5 has also been proved since apoA5 is associated with cytoplasmic lipid droplets (LDs) and affects intrahepatic TG accumulation. Furthermore, since adipocytes provide the largest storage depot for TG and play a crucial role in the development of obesity, we could infer that apoA5 also acts as a novel regulator to modulate TG storage in adipocytes. In this review, we focus on the association of gene and protein of apoA5 with obesity and metabolic syndrome, and provide new insights into the physiological role of apoA5 in humans, giving a potential therapeutic target for obesity and associated disorders.

## Background

Obesity, defined as having a body mass index (BMI) of greater than 30 kg/m^2^, is associated with a series of health problems which are always grouped together as metabolic syndrome, including type 2 diabetes, dyslipidemia and fatty liver disease [1]. The prevalence of obesity in both developed and developing countries has risen markedly, posing serious risks to future health of humans and leading to a high mortality in the general population all over the world [2]. With the in-depth study, it is now clear that the mechanism of obesity is due to an imbalance of energy intake and consumption [3], and several nutraceuticals and functional food ingredients that are beneficial to vascular health may represent useful compounds that are able to reduce the overall cardiovascular risk induced by dyslipidemia [1]. The hallmark of obesity is excessive accumulation of triglyceride (TG) in adipose tissue [4, 5]. Adipocytes, which are the predominant cell type of this tissue, have been shown to be not only a huge repository of excess energy in form of fat [6] but also a significant source of many metabolites, cytokines and hormones named adipokines under physiological and pathological conditions [7]. In obese state, adipocytes are enlarged and involved in the inordinate regulation of lipid metabolism [8]. The adipocytes isolated from obese mice has an obviously increased in volume, accompanied by various metabolic abnormalities such as cytokine secretion disorders [9] and insulin resistance [10]. In humans, dysfunctional adipocytes have been considered as an important pathophysiological basis of obesity and its related chronic metabolic diseases.

In recent years, a newly discovered apolipoprotein has been proved to be closely related to the occurrence of obesity and metabolic syndrome. Apolipoprotein A5 (apoA5), which was first described in 2001 and considered as a member of the apolipoprotein superfamily, plays an important role in modulating TG metabolism [11]. Increasing evidence indicate that apoA5 is a potent regulator of TG despite its low concentration in plasma. Mice lacking APOA5 gene had four times greater plasma TG levels than control group while overexpressing APOA5 gene presented a 66% decrease in plasma TG levels [11–13]. Furthermore, the variants of APOA5 gene in human of different ethnic groups not only influence plasma TG concentration but also have an association with the prevalence of obesity or metabolic syndrome. Consistently, lower plasma level of apoA5 was found in obese subjects and was inversely correlated with BMI in humans [14, 15], suggesting that decreased plasma apoA5 levels may have correlation with pathophysiology of obesity. However, the underlying mechanisms are still ambiguous and cannot fully explained by the controlling effect of apoA5 on plasma TG level. On the other hand, it is noteworthy that apoA5 could also modulate TG storage in hepatocytes [16], indicating a crucial intracellular role of apoA5. Since adipocytes provide the largest storage depot for TG within the lipid droplets (LDs) in humans, studies have also illuminated apoA5 could target to adipocytes and regulate intracellular TG storage [17, 18], thus exerting its beneficial effect against obesity. In this review, we focus on the association of APOA5 gene polymorphisms with obesity and the metabolic syndrome, and the potential mechanisms by which apoA5 may contribute to individual susceptibility to these conditions.

## Structural features and tissue distribution of apoA5

The results of structural and functional studies may help us to understand the basic structure of the apoA5 and its tissue distribution. The human APOA5 gene, exclusively expressed by the liver [19], contains four exons and is located on the long arm of human chromosome 11 adjacent to the APOA1/APOC3/APOA4 gene cluster [11, 19]. Previous studies revealed that apoA5 protein could be synthesized into plasma by liver at a fairly low level, and the concentration of it ranged from 5.4 to 455.6 ng/ml in healthy humans [20]. However, recent studies found that a small amount of APOA5 was expressed in human intestine [21], but the physiological functions of this APOA5 gene are still more ambiguous.

The human newly synthesized apoA5 protein consists of 366 amino acids is associated with high-density lipoproteins (HDL), very low density lipoproteins (VLDL) and chylomicrons (CM) [22]. The mature apoA5 protein consists of 343 amino acids and is a highly hydrophobic protein with rich in α-helix content [23]. Structure-function studies have indicated that apoA5 contained two independently folded domains, namely a positively charged sequence consisting of the amino acids at positions 186–227, which become a receptor binding domain and could mediate the interaction between apoA5 and heparin or LDL [24]. The C-terminal domain (293–343) of apoA5 protein mediates its binding to lipids [23]. In addition, apoA5 also contains a highly hydrophobic lipid-binding domain (161–181), which binds apoA5 to the surface of LDs [25, 26].

## Expression regulation of APOA5 gene and protein

Several nuclear receptors have been confirmed in upregulation of APOA5 gene. Pharmacological studies have shown that APOA5 was a newly discovered target gene of peroxisome proliferator-activated receptor (PPAR) family. Activated PPAR-α could bind to the corresponding site on the promoter of APOA5 gene, thereby increasing the transcriptional expression of APOA5 gene. This progress could induce a significantly reduction of plasma TG content [27, 28]. From this point, since fibrates could specifically activate PPAR-α, the mechanism of fibrates in lowering TG and inhibiting dyslipidemia could be explained, at least partly, by up-regulating APOA5 expression [29]. Nevertheless, PPAR-γ could not directly interact with apoA5 gene or protein [27]. Researchers have confirmed that PPAR-γ was decreased in APOA5 transgenic mice. Using rosiglitazone, a specific agonist of PPAR-γ, although the gene expression of APOA5 was up-regulated by nearly 70%, the plasma concentration of apoA5 protein was decreased compared to the control group. Therefore, the researchers believed that although both PPAR-α and PPAR-γ belong to the PPAR family, their effects on APOA5 expression and function were quite different [30, 31].

Secondly, farnesoid X receptor (FXR) and retinoic acid receptor-related orphan receptor (ROR) have also been confirmed to upregulate APOA5 gene expression. Using chenodeoxycholic acid, one of the FXR agonists, the researchers found that plasma concentration of apoA5 in mice was increased obviously. However, when mice knocked out FXR gene, the plasma content of apoA5 decreased [32]. On the other hand, after transfection of ROR-α into HepG2 cells, researchers found a significant increase of APOA5 gene expression, and among four subtypes of ROR-α, only α1 and α4 could transcriptionally up-regulate the expression of APOA5 [33]. Meanwhile, the promoter activity of APOA5 gene was significantly enhanced by transfection of ROR-α, and the researchers demonstrated that the specific structure which was in charge of the transcriptional regulation of APOA5 gene expression by ROR-α were three motifs composed of AGGTCA [34].

By contrast, other factors have also been confirmed to down regulate expression of APOA5 gene. T0901317, a specific receptor agonist of liver X receptor (LXR), could significantly decrease the gene and protein expression level of apoA5 in APOA5 transgenic mice. Meanwhile, co-transfection of T0901317 with sterol regulatory element-binding protein 1c (SREBP-1c) could also inhibit APOA5 gene promoter activation, whereas when SREBP-1c was silenced, the effect of T0901317 in down-regulating APOA5 gene expression was weakened [35]. These results revealed that LXR reduce the gene expression of APOA5 through the activation of SREBP-1c. Additionally, recent studies have also shown that insulin could lead a substantially lower expression of APOA5 gene and promote the intracellular TG accumulation in a dose-dependent manner in human hepatic cells, and similar results were also obtained in insulin resistant rats [15]. Notably, patients with obesity or diabetes have lower plasma apoA5 levels compared with the healthy controls, and experimentally-induced hyperinsulinemia also reduced plasma APOA5 levels in healthy men [36]. Furthermore, the researchers used the genomics method and found insulin could activate the phosphatidylinositol 3-kinase (PI3K) signaling pathways and the p70-S6 kinases (p70-S6K) pathways to down-regulated APOA5 gene expression [37]. Interestingly, in contrast to insulin, glucose could promote the transcriptional expression of APOA5 by activating the progress of de-phosphorylation of phosphorylation of upstream stimulatory factors (USFs) [15, 38], and researchers have verified that glucose could promote the binding of USFs (USF1 and USF2) to the functional E-box motif and subsequently facilitate the transcriptional expression of APOA5 gene [39].

## APOA5 gene polymorphisms on obesity and the metabolic syndrome

### APOA5 single nucleotide polymorphisms (SNPs) and obesity

APOA5 is one of the strongest regulators of plasma TG concentrations and has been linked to obesity. Nevertheless, its mechanisms of action are poorly characterized. Evidence have established an association between the presence of APOA5 gene SNPs and the risk of obesity. As Corella reported in 2007, they examined the interaction between the -1131 T > C and c.56C > G (S19 W) of APOA5 SNPs and the nutrient intake (total fat, carbohydrate, and protein) in their relation to the BMI and obesity risk in 1073 men and 1207 women participating in the Framingham Offspring Study. By analysis, they found a statistically significant interaction between the -1131 T > C SNP (not the c.56C > G) and total fat intake [40]. In subjects homozygous for the -1131 T major allele, BMI increased as total fat intake increased. Conversely, this increase was not present in carriers of the -1131C minor allele. Moreover, they found -1131C minor allele carriers had a lower obesity risk in the high fat intake group. The data showed that -1131 T > C SNP could modulate the effect of fat intake on BMI and obesity risk in both men and women [40].

Recently, Sanchez-Moreno have confirmed the interaction between -1131 T > C and dietary fat could modulate TG concentrations and anthropometric measures in overweight and obese participants. They recruited 1465 participants from a Spanish population with obesity and found the TG concentration of the patients carried -1131 T > C SNP were higher in carriers of the minor allele. Moreover, they found the participants with homozygous for the -1131 T major allele had a positive association between fat intake and obesity, whereas in those carrying the -1131C minor allele, higher fat intakes were not associated with higher BMI [41]. Consistent with findings, Horvatovich determined four haplotype-tagging polymorphisms (− 1131 T *>* C, IVS3 + 476G *>* A, c.1259 T *>* C and c.56C *>* G) and verified the frequency of major naturally occurring haplotypes of APOA5 in obese children [42]. In the pediatric patients, they demonstrated -1131 T *>* C, IVS3 + G476A and c.1259 T *>* C variants had an association with elevated TG concentration, both in obese patients and in the controls. The prevalence of the APOA5*2 haplotype (containing the minor allele of -1131 T *>* C, IVS3 + G476A and c.1259 T *>* C SNPs together) was 15.5% in obese children and 5.80% in the controls, and this haplotype confers susceptibility for development of obesity [43]. By contrast, the APOA5*4 haplotype (with -1131C alone) did not show similar associations, while the APOA5*5 haplotype (c.1259 T *>* C alone) could be protective against obesity [43, 44].

Interactions between APOA5 and other genes with obesity risk have also been studied. Since apoA5 protein and LPL proteins interact functionally to regulate lipid metabolism, and SNPs for each gene were associated with obesity risk, so evaluating gene combinations may be more effective than single SNP analyses in identifying genetic risk. Nevertheless, insufficient minor allele frequency (MAF) often limits evaluations of potential epistatic relationships. Interestingly, Smith and colleagues examined relationships between LPL m107 and c.56C *>* G and lipid and anthropometric measures in Caribbean origin Hispanics (*n* = 1019) in the Boston metropolitan area, and they found a higher BMI, waist and hip circumference in carriers with LPL m107 and the c.56C *>* G minor allele [45]. Additionally, the risk of extreme obesity (BMI ≥ 40 kg/m2) was about fourfold increased for minor allele carriers for both SNPs, suggesting that c.56C *>* G and LPL m107 could interact to modulate obesity [46]. Additionally, Chen et al. also investigated the associations of APOA1 and APOA5 SNPs and their haplotypes with some age-related diseases, as well as with lipids and proteins serum levels in a cohort from a Brazilian Elderly Longitudinal Study (EPIDOSO). They found that C-allele of APOA5 SNPs was associated with higher HDL and the GC haplotype, which was composed of the G-allele of APOA1 SNPs and the C-allele of APOA5 SNPs (summarized in Table 1), was significantly associated with obesity, with higher glycated hemoglobin, and fasting glucose [47]. Thus, these results showed that these SNPs were involved in the development of obesity and in alterations of lipids and proteins serum levels in a Brazilian population, and the present findings may also clarify the role of these SNPs and their haplotypes in lipid metabolism.

**Table 1:** The single polymorphisms of APOA5 gene and the association with obesity and metabolic syndromes

Gene	SNP/position	Association with diseases
APOA5	-1131 T > C	CAD
		Elevated plasma TG
		Elevated LDL-TG
		Decreased HDL-C
		Elevated VLDL-C
		Decreased LDL particle size
		Elevated TG after fasting
		Elevated postprandial TG
		Elevated postprandial VLDL
		Higher dense postprandial LDL
		Elevated postprandial CRP
		Elevated total cholesterol
		Elevated BMI
		Elevated fat intake
		Lower obesity risk
	c.1259 T *>* C	Elevated plasma TG
		Protective against obesity (alone)
	c.56C *>* G	Higher BMI, waist and hip circumference
		Extreme obesity (carried with LPL m107)
		Decreased HDL
	c.3A > G	Elevated metabolic syndrome risk
	c.553G > T	Elevated metabolic syndrome risk
	c.724C > G	Elevated plasma TG
	IVS3 + 476G *>* A	Elevated plasma TG

*CAD* coronary artery disease, *TG* triglyceride, *LDL* low density lipoprotein, *VLDL* very low density lipoprotein, *LDL-C* low density lipoprotein cholesterol, *CRP* C-reactive protein, *LPL* lipoprotein lipase, *SNP* single nucleotide polymorphism, *BMI* body mass index

### APOA5 SNPs and metabolic syndrome

Obesity has been identified to promote the development of metabolic syndrome, so we could speculate APOA5 SNPs may also have potential impact on the metabolic syndrome. Indeed, APOA5 SNPs were reported to be associated with two components of metabolic syndromes: higher TG levels and lower HDL levels.

Early in 2007, most studies about the physiological effects of APOA5 SNPs have focused on -1131 T *>* C and the results have already demonstrated an independent risk for -1131 T *>* C SNP in the development of metabolic syndrome. Niculescu and colleagues used a case-control design to determine the association of two APOA5 gene SNPs in a group of urban Romanian subjects with metabolic syndrome. They assayed -1131 T *>* C SNP for 279 subjects and found a high frequency for -1131 T *>* C distributed in overweight subjects. The BMI and TG levels were higher in metabolic syndrome patients carried C allele at the -1131 T *>* C SNP, however, these C allele homozygotes patients presented lower HDL-C and higher glucose levels compared to subjects with the native gene [48]. Consistent with these findings, Maasz and colleagues studied a total of 421 individuals (211 metabolic syndrome patients and 210 controls) and demonstrated in the group of metabolic syndrome patients, the prevalence of the -1131 T *>* C SNP was increased compared to the healthy controls. In both groups, the TG levels and the risk of metabolic syndromes were significantly increased approximately threefold of patients with the -1131C compared to the subjects with homozygosity for the major T allele [44, 49]. More recently, Ajjemami investigated the relative contribution of commons APOA5 SNPs and haplotypes to the risk of metabolic syndrome in Moroccan patients. They genotyped APOA5 SNPs in 176 patients and 105 controls and the statistical analysis showed a significant association between -1131 T > C SNP with metabolic syndrome. The patients carried -1131 T > C SNP was associated with increased TG level, waist circumference, fasted glucose and reduced HDL levels. These data confirmed the association of -1131 T > C variants with the predisposition to metabolic syndrome [50].

In addition, several studies have also identified the link between -1131 T *>* C SNP and the risk of metabolic syndrome in Asian people [51, 52]. In 2008, Hsu used the sample population comprised 615 unrelated subjects, 18.7% of whom had metabolic syndrome, and found a significantly higher level of TG and a lower level of HDL-C in carriers of the C allele at -1131 T *>* C SNP than in the non-carriers. Even after adjusting for age, gender, smoking, and regular exercise, the -1131 T *>* C SNP carriers remained significantly associated with an increased risk of metabolic syndrome [53]. Furthermore, in 2011, a more comprehensive study was conducted by Ong, in terms of both gene coverage and sample size to investigate the associations of APOA5 gene SNPs with the metabolic syndrome in the Hong Kong and Guangzhou Chinese. They genotyped five tagging SNPs in 1330 unrelated subjects from the Hong Kong Cardiovascular Risk Factor Prevalence Study (CRISPS) cohort with follow-up after a median interval of 6.4 years, and 1952 subjects from the Guangzhou Biobank Cohort Study-Cardiovascular Disease Sub-cohort (GBCS-CVD). After analysis, the results showed that -1131 T > C SNP was associated with an approximately 50% higher risk of metabolic syndrome in both two cohorts. These results remained the same after a 6.4-year follow-up period, indicating that the association of -1131 T > C SNP with dyslipidemia could also contribute to an increased susceptibility to metabolic syndrome in the Chinese, as a result of its effect on TG metabolism [54].

Nevertheless, it should be noted that the association of -1131 T *>* C SNP with metabolic syndrome was not found in German, Austrian and Turkish populations. The ethnic differences in minor allele frequency of -1131 T *>* C SNP, from 35.3% in Japanese [55] and 28.3% in Chinese populations [52] to 12.8% in a Turkish population [56] and 7.5% in Caucasian populations [57], may explain this discrepancy, suggesting an ethnic-specific effect of genetic variants in APOA5 on the risk of metabolic syndrome.

Additional data have provided evidence for the association of other functionally relevant APOA5 gene SNPs with metabolic syndrome. A Japanese study examined 44 SNPs at 31 candidate genes and demonstrated that minor alleles at 2 of the APOA5 SNPs examined (c.3A > G and c.553G > T) were significantly associated with increased metabolic syndrome risk [55]. Additionally, Salehi et al. genotyped 116 Iranian children and adolescents with/without metabolic syndrome to explore the association of four INDELs variants of APOA5 gene with risk of metabolic syndrome and its clinical components. They identified a novel insertion polymorphism, c. *282–283 insAG/c. *282–283 insG variant. This SNP showed a significant elevation of TG levels and the risk of metabolic syndrome, revealing that the newly identified SNP might influence the susceptibility of the individuals to metabolic syndrome [58]. More recently, Oliva used pyrosequencing technology to make DNA methylation patterns of three APOA5 regions [promoter, exon 2 and CpG island (CGI) in exon 3] and followed a recruit-by-genotype strategy to study a population composed of 44 individuals with high CVD risk selected as being carriers of at least one APOA5 SNPs (− 1131 T > C and/or, c.56C > G and/or c.724C > G) compared against 34 individuals wild-type (WT) for these SNPs. They demonstrated that carriers of APOA5 SNPs had an average of 57.5% higher circulating TG levels. Among the DNA methylation patterns of APOA5 regions, exon 3 methylation showed a positively relation with TG concentration and with a lipoprotein profile associated with atherogenic dyslipidemia. The highest TG concentrations were found in carriers of at least one SNP and with a methylation in exon 3. In conclusion, CGI methylation in exon 3 of APOA5 gene, in combination with -1131 T > C, c.56C > G and c.724C > G polymorphisms (summarized in Table 1), could play an important role in the predisposition to high circulating TG levels in humans [59]. These results provided examples that combined analysis of SNPs and methylation applied to a larger set of genes would improve our understanding of predisposition to HTG.

## The mechanism of apoA5 in modulating obesity and metabolic syndrome

### The extracellular role of apoA5

As described above, very low concentrations of apoA5 (approximately 0.1% *w*/w of apoA1) in plasma could unexpectedly has a tremendous and negative impact on plasma TG homeostasis. Knockdown APOA5 gene in mice confers a four-fold increase of plasma TG levels, whereas high-expression of the human APOA5 gene in mice leads to a decrease of TG by approximately 50% [60]. Moreover, human genetic studies have identified several APOA5 SNPs affected plasma TG levels [61]. Inherited deficiency of the APOA5 gene in humans leaded to severe hypertriglyceridemia. To date, several intensive researches have confirmed the molecular basis of apoA5 on plasma TG levels and the extracellular role of apoA5 seems to be fully elucidated. Indeed, several mechanisms may account for the effect of apoA5 on modulating plasma TG removal.

On one hand, one of the potential mechanisms by which apoA5 regulates plasma TG is the direct or indirect stimulation of LPL-mediated lipolysis of TRLs and their remnants. As we know, lipolysis occurs on the luminal surface of capillaries of skeletal muscle and adipose tissues, and LPL is the rate-limiting enzyme of plasma TG removal [62]. Even though widely distributed in a variety of tissue cells in humans, LPL synthesized in muscle and adipocytes is mainly translocated to capillary endothelial cells. Existed results showed that LPL could attach to the luminal surface of capillary endothelial cells by acting on heparin sulfate proteoglycans (HSPG) to access TRLs particles and hydrolyze them [63]. Using [3H]-triolein-labeled VLDL-like particles, the researchers found that purified apoA5 protein could stimulate LPL activity in a dose-dependent manner; injection of [3H]-triolein-labeled VLDL-like particles into APOA5 transgenic mice could significantly accelerate the rate of TRLs clearance of VLDL compared with that in control group, suggesting that apoA5 could increase the effect of LPL in reducing plasma TG level [64]. To observe the role of apoA5 in stimulating the activity of LPL, researchers used APOA5 knockout mice and transfected with LPL and a group of APOA5 transgenic mice lacking LPL, and they found that TG levels was significantly decreased in APOA5 knockout mice with highly active LPL, while in the group of APOA5 transgenic mice lack of LPL, TG levels was decreased slightly [65]. These data revealed that the effect of apoA5 in lowering plasma TG level was achieved via the presence of LPL.

**Fig. 1:**
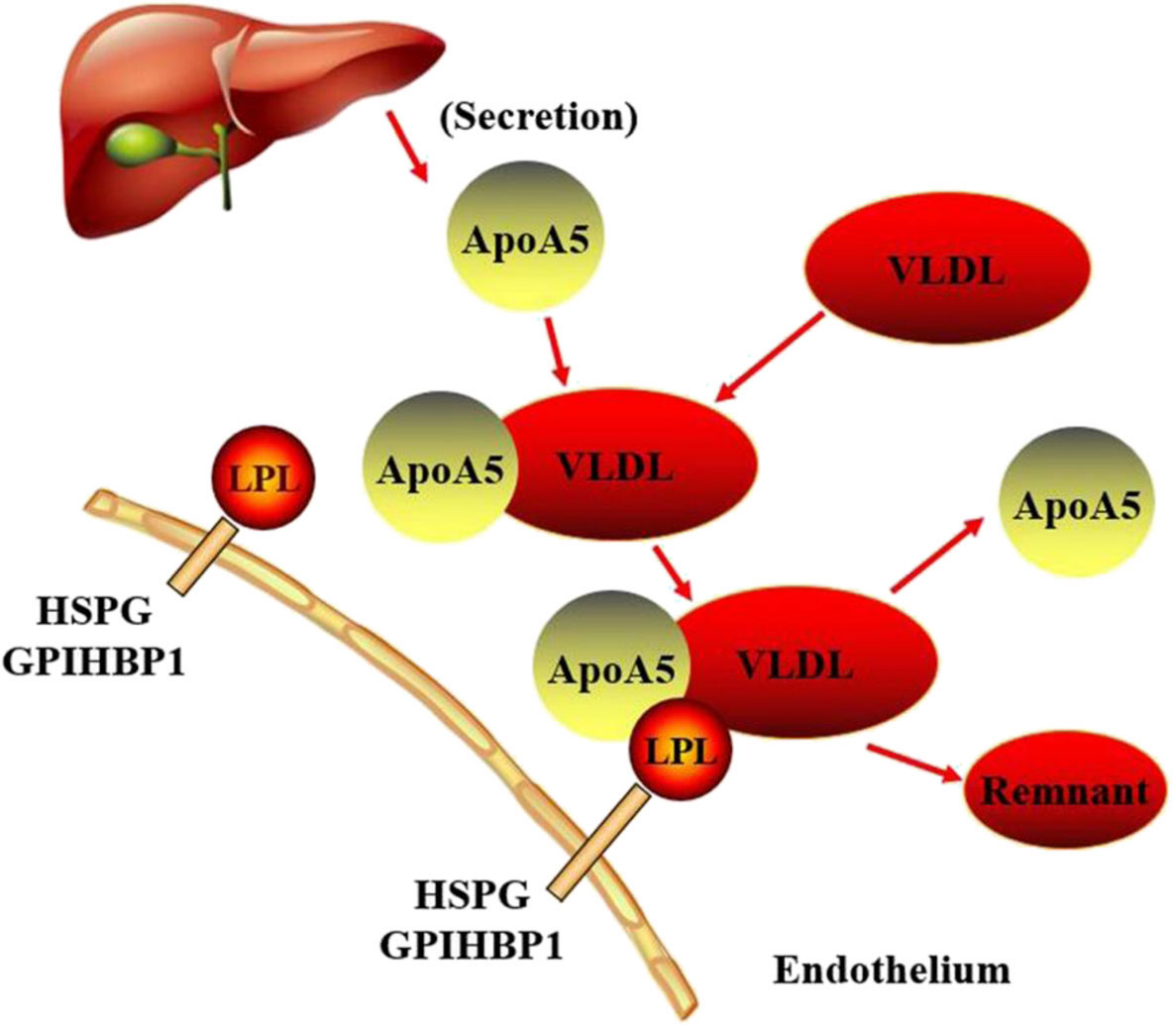
The extracellular role and the mechanisms of ApoA5. ApoA5 accelerates plasma TG hydrolysis by LPL and can be reused. See text for details

Merkel and Kluger have reported that apoA5 could accelerate plasma hydrolysis of TRLs by facilitating interaction with HSPG bound LPL but not with free LPL [66, 67]. Recent data also indicated that apoA5 could serve as a ligand for glycosylphosphatidylinositol high-density lipoprotein binding protein 1 (GPIHBP1), a novel endothelial cell surface protein, which has been postulated to serve as a platform supporting lipolytic activity [68]. Interestingly, Shu et al. found that intravenous injection of recombinant apoA5 protein significantly lowered plasma TG concentrations in APOA5-deficient mice but not in GPIHBP1-knockout mice [16, 69]. Based on these studies, it is conceivable to speculate that apoA5 promotes attachment of TRLs to endothelial cell surface HSPG or GPIHBP1 and that such interactions enhance lipolysis. On the other hand, the hypotriglyceridemic effect of apoA5 could also be explained by the regulation of TRL clearance mediate by LDL-receptor (LDLR). Numerous of studies have discovered a special positively charged region of apoA5 which was composed of 42 amino acid residues. It has been demonstrated that heparin could bind to members of the LDLR family in this special functional region [65]. Based on the findings that apoA5 can interact with both HSPG and LDLR family members via positively charged regions, and that both HSPG and LDLR family members function to mediate hepatic endocytosis of TRLs, it is reasonable to speculate that apoA5 could enhance hepatic uptake of these particles. Indeed, Grosskopf reported that removal of chylomicron remnants from plasma was impaired in APOA5-deficient mice, and uptake of remnants in liver-perfusion experiments was weakened [70, 71]. Consistent with these findings, experiments using surface plasmon resonance showed that association of apoA5 with LRP1 resulted in enhanced binding of human chylomicrons to receptor covered sensor chips (Fig. 1). Intriguingly, injection of APOA5 transgenic mice with heparin increased plasma apoA5 levels by approximately 25% indicating the existence of a heparin-releasable pool [72].

Overall, these results provide evidence for the possibility that some portion of newly secreted apoA5 protein might bind to HSPG or to receptors on the surface of hepatocytes where it could facilitate lipoprotein binding and uptake.

### The intracellular role of apoA5

Whereas a portion of hepatic-derived apoA5 is secreted into plasma and functions to facilitate LPL-mediated TG hydrolysis, another portion of apoA5 is confirmed to recover intracellularly and is associated with cytosolic LDs [73, 74].

**Fig. 2:**
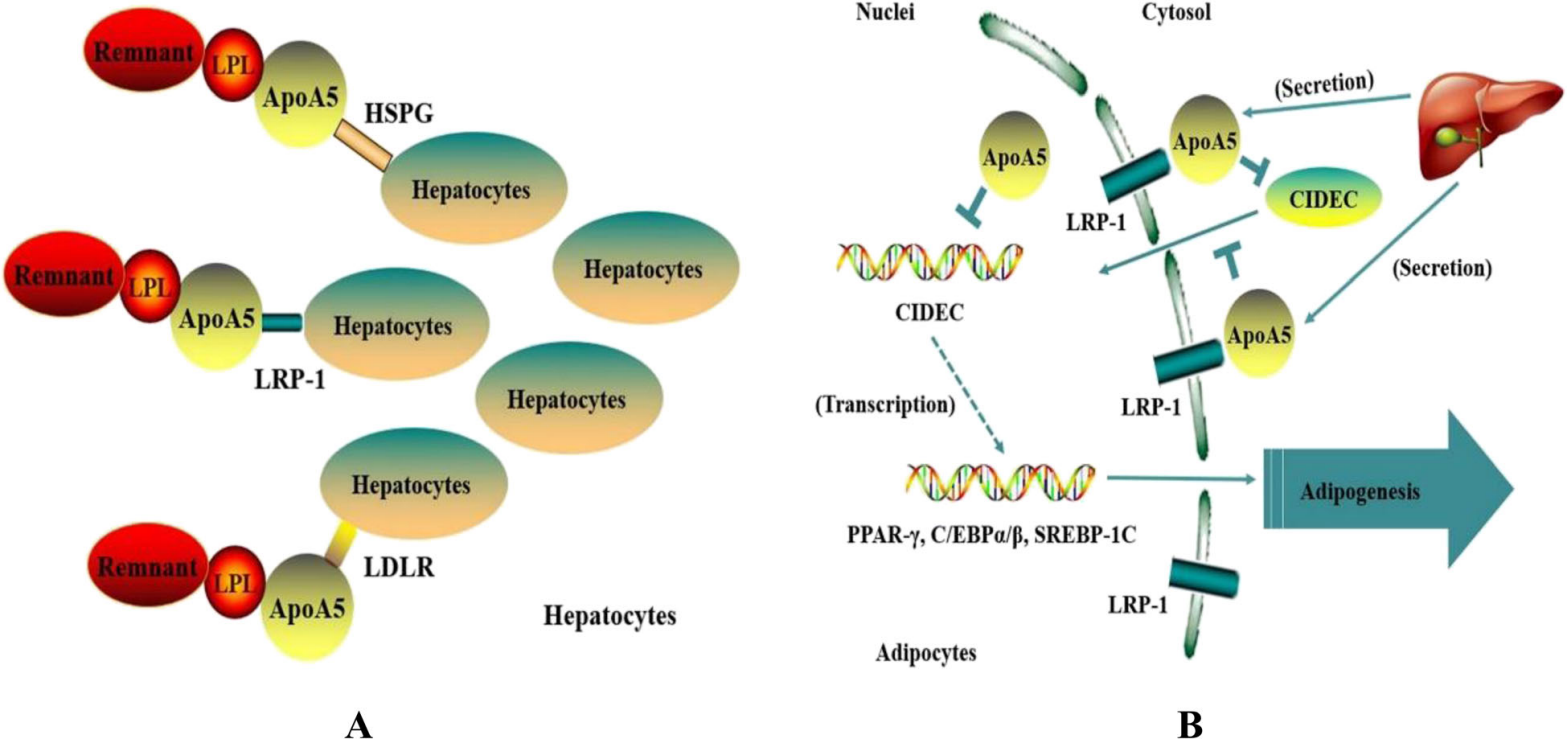
The intracellular role and the mechanisms of ApoA5 in adipocytes and hepatocytes. **a** ApoA5 may mediate receptor or proteoglycan mediated remnant uptake into the liver. See text for details. **b** ApoA5 may inhibit the progress of adipogenesis differentiation of AMSCs. See text for details

Several results have provided convincing evidence that apoA5 has a role in regulating intracellular TG metabolism. Using transfected doxycycline-inducible McA-RH7777 rat hepatoma cell line, Blade et al. found that apoA5 treatment could inhibit TG secretion, increase intracellular TG concentration and reduce VLDL particle; when stably transfected McA-RH7777 cells were treated with oleic acid, the resulting increased in TG synthesis caused a reduction in apoA5 secretion, which has been considered as a reciprocal increase in cell-associated apoA5. However, no impact on apolipoprotein B (apoB) secretion was observed [75]. These data indicated that reduced TG secretion observed upon induction of apoA5 treatment could be due to the attenuation of second-step particle maturation, essential for the formation of TG-rich VLDL. Additionally, the fact that intrahepatic apoA5 associated with LDs provides evidence that apoA5 can function like many known LD-associated proteins, such as perilipin and adipose differentiation-related protein (ADRP), to help modulate intracellular lipid metabolism. Indeed, studies with APOA5 transgenic mice revealed that apoA5 expression influences intrahepatic TG accumulation [76], and Ress et al. also demonstrated that knockdown of APOA5 in human hepatoma cells resulted in decrease in intracellular TG content (Fig. 2a) [30].

ApoA5 is a potent regulator in TG metabolism in hepatocytes, revealing a crucial intracellular function of apoA5. Since adipocytes are the largest storage depot for energy in the form of TG within the LDs in humans [77], it is reasonable to speculate that apoA5 may also target to adipocytes and regulate intracellular TG storage, providing a potential therapeutic target for obesity. Recently, Zheng and colleagues used pulse-chase experiments and found that apoA5 could be internalized into human mature adipocytes, and about 70% of the apoA5 internalized during the pulse remained intracellular within a 24-h chase [72]. Pre-incubation with heparin and the receptor-associated protein, both of which could prevent the apoA5 interaction with members of the LDLR gene family, markedly reduced the uptake of apoA5 by 61 and 52%, respectively; meanwhile, using adipocytes with LRP1 knockdown also resulted in a decrease in internalized apoA5 content [72]. Thus, these data revealed that LDLR family members, at least partly, play an important role in inducing apoA5 internalized by adipocytes. In addition, the group also confirmed that the internalized apoA5 was co-localized with LDs in adipocytes by using confocal microscopy and that apoA5 could significantly decrease intracellular TG storage and the expression of the LDs-associated proteins such as cell death-inducing DNA-fragmentation-factor like effector C (CIDE-C) and perilipin, leading to a generally smaller LDs in apoA5-treated cells compared with those in control cells [72, 78]. Furthermore, the adipocytes intervened with apoA5 also presented an obviously increased in lipolysis activity and the gene expression of uncoupling protein 1 (UCP1) [78]. As UCP1 is the brown adipose tissue (BAT)-specific gene and is recognized as the molecular effector of thermogenesis in brown adipocytes [79], these results suggested that decreased TG accumulation in adipocytes induced by apoA5 may be associated with enhanced lipolysis and energy expenditure.

As demonstrated previously, CIDE-C has been identified as a novel LDs-associated protein in controlling diverse metabolic processes [80, 81]. To date, researchers focus on the crucial role of CIDE-C in promoting AMSCs differentiation. The latest results showed the level of CIDE-C gene could be significantly up-regulated during the differentiation of AMSCs, and silencing CIDE-C could induce AMSCs losing its ability to differentiate into mature adipocytes [82]. The adipose tissue has abundant adipose-derived mesenchymal stem cells (AMSCs), which can differentiate into mature adipocytes by imbalance between energy intake and expenditure, and the excessive adipogenesis differentiation of AMSCs can lead hyperplasia of adipocytes and promote the pathophysiological development of obesity [83]. Actually, the marked effect of apoA5 on modulating intracellular TG storage and preventing human mature adipocytes from hypertrophy has sparked our interest to investigate whether apoA5 could act as an important regulator for the AMSCs (Fig. 2b). This interesting phenomenon may help us to establish a link of interaction between apoA5 and CIDE-C, but the mechanism of apoA5 in decreasing the expression of CIDE-C is still ambiguous and we need further research.

## Conclusions

Several studies have given evidence for the interaction between APOA5 SNPs and increased risk of obesity and metabolic syndromes, suggesting that the genetic variability of APOA5 plays an important role in modulating lipid metabolism, and the mechanisms of action seems to be clear. Firstly, apoA5 has been verified to target lipoproteins with HSPG-bound or GPIHBP1-bound LPL, promoting the efficiency of lipoprotein hydrolysis. Secondly, apoA5 facilitates hepatic uptake of TRLs and their remnants through direct interaction with HSPG and meanwhile, apoA5 could also reduce the VLDL-TG production in liver as a result of impaired second-step lipidation of VLDL, leading to a lower TG concentration in plasma. In humans, apoA5 could be internalized by human adipocytes primarily via binding to LRP1, and the uptake of apoA5 was attenuated in obese adipose tissues and in cultured adipocytes with hypertrophy or insulin resistance. In addition, decreased TG accumulation in human adipocytes induced by ApoA5 intervention may be associated with enhanced lipolysis and energy expenditure, which may result from reduced expression of CIDE-C and perilipin. These findings may provide a greater understanding of the roles of apoA5 in regulating the intracellular TG metabolism of adipocytes.

Furthermore, under hypertrophied and insulin resistant conditions, attenuated endocytosis of apoA5 by adipocytes may lead to excessive augmentation of TG storage and abnormal metabolism of adipocytes, which promotes the development of obesity. As a novel regulator of lipid storage in adipocytes, apoA5 may serve an important role in whole body energy homeostasis and may be a potential therapeutic target for the treatment of obesity and metabolic syndromes. Besides, as nutraceuticals and functional food ingredients have been verified beneficial to vascular health may represent useful compounds that are able to reduce the overall cardiovascular risk induced by dyslipidemia by acting parallel to medicine or as adjuvants in case of failure or in situations where medicine cannot be used, using apoA5 and nutraceuticals as a combined therapy will become a new treatment.

## Abbreviations

ADRP: Adipose differentiation-related protein
AMSCs: Adipose-derived mesenchymal stem cells
ApoA5: Apolipoprotein A5
ApoB: Apolipoprotein B
BAT: Brown adipose tissue
BMI: Body mass index
CAD: Coronary artery disease
CIDE-C: Cell death-inducing DNA-fragmentation-factor like effector C
CM: Chylomicron
CRISPS: Hong Kong Cardiovascular Risk Factor Prevalence Study
CRP: C-reactive protein
CVD: Cardiovascular disease
FFA: Free fatty acids
FXR: Farnesoid X receptor
GPIHBP1: Glycosylphsphatidylinositol-anchored high-density lipoprotein binding protein 1
HDL: High density lipoprotein
HSPG: Heparin sulfate proteoglycans
LDL: Low density lipoprotein
LDLR: LDL-receptor
LDs: Lipid droplets
LPL: Lipoprotein lipase
LXR: Liver X receptor
MAF: Minor allele frequency
p70-S6K: p70-S6 kinases
PI3K: Phosphatidylinositol 3-kinase
PPAR: Peroxisome proliferator-activated receptor
ROR: Retinoic acid receptor-related orphan receptor
SNP: Single nucleotide polymorphism
SREBP-1c: Sterol regulatory element-binding protein 1c
TG: Triglyceride
UCP1: Uncoupling protein 1
USFs: Upstream stimulatory factors
VLDL: Very low density lipoprotein
